# Effectiveness of Systematic Periodontal Treatment in Male HIV-Infected Patients after 9 Years: A Case Series

**DOI:** 10.1155/2018/4135607

**Published:** 2018-08-13

**Authors:** Rainer A. Jordan, Adrian Lucaciu, Katharina Schaper, Hans-Peter Jöhren, Stefan Zimmer

**Affiliations:** ^1^Department of Operative and Preventive Dentistry, Faculty of Health, School of Dentistry, Witten/Herdecke University, Witten, Germany; ^2^Department of Periodontology, Faculty of Health, School of Dentistry, Witten/Herdecke University, Witten, Germany; ^3^Institute of Medical Biometry and Epidemiology, Faculty of Health, School of Medicine, Witten/Herdecke University, Witten, Germany; ^4^Department of Oral Surgery, Faculty of Health, School of Dentistry, Witten/Herdecke University, Witten, Germany

## Abstract

**Objective:**

To investigate effectiveness of systematic periodontal treatment in the long term in HIV-infected patients undergoing highly active antiretroviral treatment.

**Methods:**

Longitudinal, prospective, open-label case series over a period of nine years. Periodontal treatment was performed by scaling and root planing and supportive periodontal care (SPC) at regular intervals. To measure effectiveness, reductions of pocket probing depths were defined as primary study endpoint.

**Results:**

During the study period, there was a proportional increase in periodontal pockets ≥4 mm of +53% and in pockets ≥ 6 mm of +100%. Mean pocket depth reductions on patient's level were, however, 0.4 mm nine years after scaling and root planing and supportive periodontal care (*p*=0.180). No teeth were lost during the observation period.

**Conclusions:**

In terms of best evidence available, it is concluded that systematic periodontal treatment including SPC is effective in virologically controlled HIV infection and can be performed in dental practice safely.

## 1. Introduction

Highly active antiretroviral therapy (HAART) has significantly changed the characteristics of chronic periodontitis in human immunodeficiency virus- (HIV-) infected individuals, but only little evidence is available about its long-term treatment outcomes [1].

Historically speaking, there is a strong diagnostic relationship between dentistry and HIV disease: as early as 1981, researchers had successfully identified the primary oral symptoms of AIDS (acquired immunodeficiency syndrome)—Kaposi's sarcoma [2]. Oral hairy leukoplakia was further identified as the only pathognomonic oral manifestation of HIV infection [3]. Before effective antiretroviral treatments for HIV became available, dentists were typically confronted with acute oral manifestations of the disease, such as necrotizing ulcerative periodontitis [4]. Since the advent of highly active antiretroviral therapy, or HAART, both clinical presentation and prognosis of HIV infection have changed dramatically and have led to a significant reduction in mortality as well as improved quality of life [5, 6]. Improved (access to) HAART shifted HIV infection into a chronic disease [7] so that today, patients with HIV can expect to have a near-normal life expectancy in developed countries [8].

In dentistry, therapeutic objectives have changed as a result of these developments, too. Highly acute periodontal diseases have been changed to more chronic forms of the disease including needs for lifelong supportive periodontal care [4]. While HAART turns HIV into a manageable disease, treatment does not lead to eradication of the virus. The human immunodeficiency virus is known to interfere with the host's immune system at different stages of both cell-mediated and humoral immune responses. With periodontal respect, at least four mechanisms are of relevance: (i) limited chemotaxis of neutrophilic granulocytes in the initial phase of periodontal bacterial infection [9, 10], (ii) dysfunction of macrophages with CD4 surface markers in the succeeding phase of phagocyte [11], (iii) disorganization of gingival B- and T-lymphocyte activity and their protective humoral products [12], and (iv) direct toxicity of certain antiretrovirals on hematopoiesis [13]. Therefore, there is potential for a long-term HIV-mediated effect on chronic periodontitis—even in spite of HAART.

Clinically, nonsurgical periodontal treatment in terms of scaling and root planing was shown to be an effective treatment procedure, and transient bacteraemia was not associated with clinical problems in patients undergoing HAART; pocket depth reductions could be observed in comparable dimensions in HIV-infected patients compared to non-HIV-infected patients within one year after treatment and consecutive supportive periodontal care in a comparative study [14]. Even in different preexisting periodontal diseases, supportive periodontal care after nonsurgical treatment led to stable periodontal conditions in HIV-infected patients in the short term [15].

Given the fact that chronic periodontitis is a lifelong disease and that oral hygiene adherence and supportive periodontal care are key factors for a sustained periodontal treatment success [16, 17], we were interested in its long-term treatment outcomes in HIV infected patients in the HAART era. It was therefore the purpose of this study to investigate effectiveness of systematic periodontal treatment including supportive periodontal care in the long term in HIV-infected patients undergoing HAART with chronic periodontitis in a controlled case series. We hypothesized that periodontal status improved by reduction of mean periodontal pocket depth on patient's level.

## 2. Study Population and Methodology

This was a longitudinal, prospective, open-label case study conducted in Germany over a period of nine years. The study was executed between 2000 and 2012. Systematic periodontal treatment in this study was defined as nonsurgical anti-infective periodontal treatment by scaling and root planing and subsequent supportive periodontal care at regular intervals. The study was approved by the Witten/Herdecke University Ethical Review Committee and registered in the German Clinical Trials Register (DRKS-ID: DRKS00000097). The methods of this case series were carried out according to the approved protocols. All subjects gave written informed consent before any study-related procedures were done.

### 2.1. Study Settings and Sample Size

As 95% of dentists in Germany work in dental practices [18], the setting of the study should represent common dental treatment conditions and report effectiveness data achievable in everyday dental care. Anti-infective periodontal treatment was therefore planned and performed under the premises of the German statutory health insurance conditions [19]. This includes scaling and root planing in all teeth with pocket probing depths deeper than 3.5 mm covered by statutory health insurance. Subsequent supportive periodontal care, however, is to be paid by the patient. To achieve our goal of aggregating data of long-term periodontal treatment outcomes with vulnerable patients and self-paid supportive periodontal care, a strict participant recruitment under the premises of a randomized controlled trial appeared to be unrealistic. Our study participant recruitment was therefore as follows: all HIV-infected patients undergoing HAART that were referred for periodontal treatment within two years (December 2000 to November 2002) to a specialist in periodontology in Berlin, Germany, were screened and interviewed whether they would participate in this study.

### 2.2. Study Population

**Figure 1:**
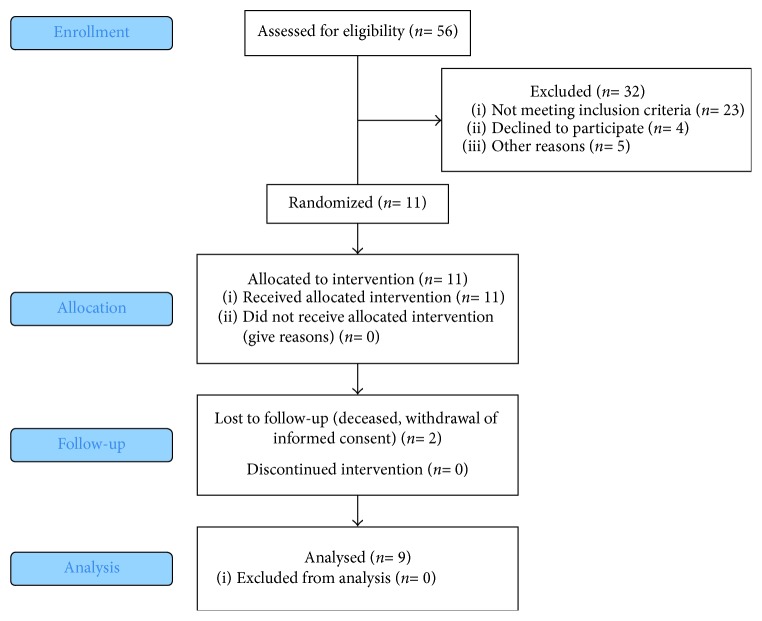
Participant study flow.

Eleven out of 56 HIV-infected patients agreed to participate, and they met the further, research-based inclusion and exclusion criteria: subjects were male adults, HIV-serotype-1 infected, underwent highly active antiretroviral therapy (HAART), and presented a sustained immune reconstitution so far. As one patient deceased and one patient withdrew study participation, nine subjects could be reevaluated after nine years. Immune reconstitution was observed by CD4 cell counts and HIV viral load. CD4 cell counts and viral load are considered to be the best prognostic markers for HIV disease progression [20]. CD4 cell counts are used for clinical classification of patients according to the CDC scheme [21]. HIV viral load indicates viral activity in the host and is a marker for successful HAART therapy [22]. Participants were between 30 and 50 years of age, showed at least 20 natural teeth, and had to be treatment-naïve concerning chronic periodontitis. As mentioned before, chronic periodontitis was defined according to the rules of the German statutory health insurance; that is, patients had to present at least three teeth with pocket probing depths deeper than 3.5 mm. In fact, participants demonstrated at least four teeth with pocket probing depths of at least 4 mm. Exclusion criteria were additional systemic risk factors associated with periodontitis such as diabetes mellitus and medication on a regular basis except for HAART. Smoking behavior was recorded. The participant flow is demonstrated in Figure 1.

### 2.3. Study Endpoints

To measure the effectiveness of systematic periodontal treatment, reductions of pocket probing depths were defined as primary study endpoint (a).

Secondary study endpoint was tooth loss (b). Further secondary study endpoints were (c-i) the plaque index (PI) according to Silness and Löe [23] to determine the extent of the oral biofilm and (c-ii) the gingival index (GI) according to Löe and Silness [24] to determine the extent of gingivitis. Both plaque and gingival indices were recorded at the follow-up examination to gather indicators of individual oral hygiene measures and effectiveness.

While analyzing the study data, new standards for reporting periodontitis prevalence and severity were published suggesting that the calculation of mean pocket depth (differences) should be accompanied by responder analyses to demonstrate the portions of not successfully treated periodontal pockets [25]. Therefore, the biometrical analysis respects the recent recommendations as far as supported by the data collected.

### 2.4. Interventions

Active periodontal treatment was followed by a standardized procedure for nonsurgical anti-infective therapy with repeated appointments of professional tooth cleaning, oral hygiene instructions, and motivation as well as instrumental removal of supragingival plaque deposits by a dental hygienist at first. A plaque index score of 0.25 was intended to achieve prior to scaling and root planing. Scaling and root planing in all teeth of pocket probing depths greater than 3.5 mm was executed under local anesthesia using the Gracey curette set at weekly intervals, one quadrant per session, by a periodontologist. Six weeks postoperatively, supportive periodontal care (SPC) started in a four-month interval in the first year for all participants. Afterwards, treatment intervals were determined on individual risk assessment according to Lang and Tonetti [26] meaning an interval of supportive periodontal care of 4 month because of HIV seropositive risk status. Supportive periodontal care consisted of instrumental removal of supragingival plaque deposits by a dental hygienist, professional tooth cleaning, and oral hygiene instructions and motivation. Subgingival debridement was repeated in case of re-inflammation and pocket bleeding by the dentist.

Before the anti-infective periodontal treatment, study-related measurements were performed at baseline (= *t*_0_) with a PCP12 periodontometer (Hu-Friedy, Tuttlingen, Germany). Pocket probing depths were measured in four sites per tooth and rounded up to the next whole millimeter. Nine years later (= *t*_1_), subjects were requested to attend a study-related follow-up appointment. An investigator who was not part of the supportive periodontal care program carried out the same measurements compared with *t*_0_.

### 2.5. Statistical Analysis

All completed subjects were evaluated for study-related endpoints. Qualitative endpoints were tested with the McNemar test, and quantitative variables were tested with the sign test, both with a two-sided significance level of *p* ≤ 0.05. Due to the special procedure and forms of application of anti-infective periodontal treatment in German statutory health insurance, the simultaneous documentation of pocket probing depths and gingival recessions in all measured tooth sites was not provided. Therefore, loss of attachment could not be calculated. IBM SPSS software (International Business Machines Corporation, Armonk, USA), version 20, was used for computing statistical analysis.

## 3. Results

The demographic and clinical characteristics are presented in Table 1.

Table 2 shows the primary study endpoint: (a) reductions in pocket probing depths. The prevalence of periodontal pockets ≥4 mm and ≥6 mm was 9.1% and 0.9% at *t*_0_ and was 14.0% and 1.8% at *t*_1_. During the study period, there was an increase in pockets ≥4 mm in the test group of +53.8% and in pockets ≥6 mm of +100.0%. The mean pocket probing depth was 2.7 mm at *t*_0_ and 2.3 mm at *t*_1_ (*p*=0.180) with mean pocket depth reductions on patient's level of 0.4 mm, expressed as difference (Δ) between *t*_1_ and *t*_0_.

The secondary study endpoint ((b) tooth loss) is shown in Table 2. During the study period, statistically no tooth loss occurred.

Secondary study endpoints ((c-i) plaque index (PI); (c-ii) gingival index (GI)) are shown in Table 3. Plaque index was 0.26, and gingival index was 0.23.

## 4. Discussion

This longitudinal case series aimed at evaluating the effectiveness of systematic periodontal treatment including supportive periodontal care for nine years in HIV-infected patients with chronic periodontitis under daily practice conditions. Mean pocket probing depth reductions in HIV-infected subjects demonstrated no statistically significant differences. Therefore, we reject the study hypothesis. However, detailed analyses of the data according to recent recommendations [25] demonstrated interesting insights and allow for differentiated interpretations. When calculated the patient's mean pocket depths, no statistically significant differences were observed over time. However, focusing only diseased sites—prevalences and proportions—an increase of periodontitis sites became apparent (Table 2). The increase of pockets with probing depths ≥4 mm reached +41% and of pockets with probing depths ≥6 mm (+63%). Therefore, it could be speculated that HIV infection might have an influence in the long-term progression of chronic periodontitis, even in the HAART era, and even under a sustained immuno-reconstitution that was observed in our patients over time. On the contrary, it must be stated that baseline prevalence of probing pocket depths ≥6 mm was quite low. Therefore, it is questionable which improvements of probing depths in these pockets could have been expected. From a clinical point of view, it is favorable to clinical management that the absolute increase in probing pocket depths ≥6 mm is low, too. Instead, some increase in probing pocket depths ≥4 mm occurred in HIV-infected patients. However, the clinical management of shallow pockets during supportive periodontal care is less ambitious than of deep pockets. Taking into account that in the HIV-infected study participants no tooth loss occurred during long-term periodontal maintenance, the results appear clinically satisfying to us.

Interestingly, the extent of the oral biofilm was quite low indicating efficient personal oral hygiene measures. Accordingly, the extent of gingivitis was also low. It might be argued that this finding corresponds with the narrow interval of supportive periodontal care in HIV-infected patients. Another explanation could be that HIV infection is accompanied with blood controls and physician's appointments at regular intervals; therefore, a distinct personal awareness of chronic disease control can be supposed in that patient group [27]. It does not appear to be implausible that improved personal disease awareness might also result in improved personal oral hygiene measures. High adherence concerning oral hygiene measures in HIV-infected patients was documented in an interventional study, too, evaluating the oral hygiene benefits after a weekly performed periodontal maintenance program over a period of six months [28]. Subjects improved their gingival health from 28% at baseline to 78% indicating that intensive professional oral hygiene interventions may result in stable oral hygiene procedures at home.

Currently, about 80,000 people in Germany are living with HIV/AIDS [29]. Of them, more than 80% are male. Approximately 70% of HIV-infected persons undergo antiretroviral therapy. No data are available concerning prevalence of chronic periodontitis among this patient group. While smoking behavior in the adult population in Germany is prevalent in one third of the adult population, prevalence among people with HIV/AIDS is considerably higher with 56% [30]. Therefore, the composition of the test group in our study seems to be representative for this patient group. The absolute number of CD4 cells pose an immunological risk factor for infections, such as periodontitis, and it was calculated that this risk factor is twice as high as compared to smoking [31]. Progressive HIV infection leads to a massive decrease in CD4 cells, and this is one major reason for increased vulnerability to infectious diseases in advanced HIV infection and AIDS. CD4 cells also represent the first line of defense against periodontal pathogens. Insofar, it is plausible that effective antiretroviral treatment shifted periodontal diseases in HIV infection from acute to chronic forms.

Results from this study cannot claim to have high external validity. We are inclined to view this study as a first longitudinal observation and would therefore expect a considerable degree of bias. The chosen study design is owed to the fact that recruitment of subjects with HIV infection under the premises of a follow-up period approaching one decade was performed depending on a high adherence to supportive periodontal care. Further, eligible subjects should not have been taking antibiotics three months prior to study inclusion. In the early 2000s, however, prescription of antibiotics in HIV infection for prophylactic reasons was still common, thus reducing the potential number of eligible subjects [32]. On the contrary, no lost to follow-up over the study period increases the validity of the study. Though there were several statistical differences found in the analyses of the data, no reductions of mean pocket probing depths could be demonstrated in the primary study endpoint. Lack of statistical differences may partly be due to the small numbers of subjects.

Management of HIV infection and life expectancy increased dramatically within the last years. It was calculated that life expectancy for people with HIV infection living in a developed country with extensive access to HAART and healthcare is 75.0 years by now [33]. Therefore, studies dealing with chronic diseases in the oral cavity and their long-term dental treatment, such as periodontal diseases, should be a contemporary focus of HIV-related oral health science in developed countries. As people with HIV infection live longer, neglecting the importance of oral health may adversely affect individuals' general health and quality of life. With respect to periodontal diseases, this means a shift in focus from HIV-associated periodontal diseases like linear gingival erythema (LGE) or necrotizing ulcerative gingivitis (NUG) and periodontitis (NUP) towards chronic periodontal diseases. So far, no comparable long-term outcomes in treatment of chronic periodontitis in HIV-infected patients are available. Hofer et al. presented midterm results of supportive periodontal therapy in HIV-infected patients as compared to non-HIV-infected patients after 23 months. They found stable clinical markers as measured by pocket probing depths, attachment levels, the oral biofilm burden, and gingivitis, whereas significant improvements were observed only in non-HIV-infected controls [15]. The long-term results of our study appear to match, as the long-time mean periodontal pocket depths of our patients remained stable, though some sites with diseased periodontal pockets increased over time.

To the best of our knowledge, these are the first long-term data on the efficacy of systematic periodontal therapy in HIV-infected patients, and they appear to suggest that our established periodontal treatment techniques may be effective in the long-term treatment of this vulnerable patient group. We find these results encouraging and feel that they warrant further investigation, as part of a larger, multicenter study.

In terms of best evidence available, it is concluded that systematic periodontal treatment including supportive periodontal care is effective in virologically controlled HIV infection as compared to non-HIV-infected patients.

## Figures and Tables

**Table 1:** Demographic and clinical characteristics of the analyzed patients.

Characteristic	Baseline (*t*_0_)	Follow-up (*t*_1_)
Sex, male	9	9
Age, mean years (SD)		47.6 (4.0)
Smoking behavior		
Yes	4	5
No	5	4
HAART		
NNRTI based	7	2
Protease inhibitor based	2	7
HIV viral load,^†^ mean (SD)	20,088 (33,459)	350 (994)
CD4 counts,^‡^ mean (SD)	581 (263)	625 (294)
Follow-up, month		113.0 (10.6)

SD, standard deviation; HAART, highly active antiretroviral therapy; HIV, human immunodeficiency virus; NNRTI, nonnocleoside reverse transcriptase inhibitor; *t*_0_, the baseline examination point in time; *t*_1_, the follow-up examination point in time. ^†^HIV DNA (copies/mL); ^‡^CD4 cells (cells/*μ*L).

**Table 2:** Prevalence of at least one affected site and extent (proportion) of affected sites and teeth per mouth by degree of pocket probing depth (PPD, cutoffs ≥4 and ≥6 mm) and mean in total.

	Baseline (*t*_0_)	Follow-up (*t*_1_)	*p* value
Number of subjects (*n*)	9	9	1.0
Tooth count (mean (SD))	26.0 (3.2)	25.0 (2.8)^#^	0.063
Prevalence PPD (sites) ≥4 mm (% (SE))	9.1 (0.96)	14.0 (1.16)	<0.001
Prevalence PPD (sites) ≥6 mm (% (SE))	0.9 (0.31)	1.8 (0.44)	0.152
Proportion of sites/mouth PPD ≥4 mm (mean (SD))	9.8 (5.4)	14.0 (9.6)	0.508
Proportion of sites/mouth PPD ≥6 mm (mean (SD))	1.0 (1.5)	1.7 (2.1)	0.453
Proportion of teeth/mouth PPD ≥4 mm (mean (SD))	28.1 (15.5)	39.2 (23.3)	0.508
Proportion of teeth/mouth PPD ≥6 mm (mean (SD))	4.1 (6.1)	5.6 (5.7)	1.000
Mean PPD (mm (SD))	2.7 (0.4)	2.3 (0.5)	0.180

PPD, pocket probing depth; SD, standard deviation; SE, standard error; HIV, human immunodeficiency virus; *t*_0_, the baseline examination point in time; *t*_1_, the follow-up examination point in time. ^#^Single implant tooth replacements were clinically counted as natural teeth.

**Table 3:** Secondary study endpoints: plaque index (PI) and gingival index (GI).

Index	Follow-up (*t*_1_)
	Median (min; max)
Plaque index (PI)	0.26 (0.0; 0.9)
Gingival index (GI)	0.23 (0.0; 0.6)

## Additional Points

*Scientific Rationale for Study*. Antiretroviral treatment progress eclipsed acute HIV-associated periodontal infections and more chronic periodontitis emerged in HIV-infected patients including needs for lifelong supportive periodontal care (SPC). *Principal Findings*. This case series presents long-term data of SPC in HIV-infected patients. Periodontal probing depths in HIV-infected patients worsened over time but no teeth were lost due to periodontitis. *Practical Implications*. In terms of best evidence available, it is concluded that systematic periodontal treatment including SPC is less effective in virologically controlled HIV infection as compared to non-HIV-infected patients but can be performed in dental practice safely.
